# A Contemporary Guide of Venoarterial Extracorporeal Membrane Oxygenation in Cardiogenic Shock

**DOI:** 10.3390/jcdd12120475

**Published:** 2025-12-02

**Authors:** Vinh Q. Chau, George Kalapurakal, Teruhiko Imamura, Ben B. Chung, Sejal Loberg, Allison Beckett, Antone J. Tatooles, Nikhil Narang

**Affiliations:** 1 Advocate Heart Institute, Advocate Christ Medical Center, Oak Lawn, IL 60637, USA; 2 Section of Cardiology, Advocate Lutheran General Hospital, Park Ridge, IL 60068, USA; 3 Second Department of Internal Medicine, University of Toyama, Toyama 930-8555, Japan; te.imamu@gmail.com; 4 Section of Cardiology, Department of Medicine, University of Chicago, Chicago, IL 60637, USA; bow.chung@bsd.uchicago.edu; 5 Endeavor Health Cardiovascular Institute, Glenview, IL 60026, USA

**Keywords:** cardiogenic shock, veno-arterial extracorporeal membrane oxygenation, temporary mechanical circulatory support devices

## Abstract

Managing refractory cardiogenic shock is individualized, with few aspects considered routine or universally contraindicated. Venoarterial extracorporeal membrane oxygenation (VA-ECMO) is a temporary mechanical circulatory support strategy, providing hemodynamic stabilization and gas exchange for patients with severe cardiogenic shock. It is increasingly used as salvage therapy for advanced cardiopulmonary failure and serves as a bridge to myocardial recovery, heart transplantation, or durable mechanical support such as a left ventricular assist device. Over the past decade, VA-ECMO utilization has risen, even though robust clinical trial evidence supporting its use remains limited. Furthermore, consensus is lacking on key aspects of care, including patient selection, cannulation strategy, weaning protocols, and complication management. This review outlines a structured approach to daily VA-ECMO care, emphasizing multidisciplinary coordination and individualized patient support to optimize outcomes and mitigate complications. We also address the implications of limited trial data and highlight the need for evidence-based frameworks to guide clinical decision-making.

## 1. Introduction

Managing refractory cardiogenic shock (CS) is highly individualized, with few aspects being routine. The most common causes of CS are acute myocardial infarction (AMI-CS) or acute-on-chronic heart failure (HF-CS), but also expand to CS related to massive pulmonary embolism or in the post-cardiotomy setting. Registry-based data suggest that HF-CS is becoming more prevalent than AMI-CS [1]. Cardiac failure in these settings typically manifests as hypotension, end-organ hypoperfusion, and hypoxia. In-hospital mortality risk in all-comers with CS ranges from 30 to 50% [2,3,4]. Pharmacological interventions and temporary mechanical circulatory support devices (tMCSDs) can be utilized to stabilize hemodynamics and restore end-organ perfusion [5]. The use of tMCSDs involves balancing the likelihood of benefits with the risk of severe complications, which could result in a net neutral effect on reducing mortality risk. Patient and device selection require careful consideration of both shock phenotypes and severity to determine the most appropriate intervention.

The Society of Cardiovascular Angiography and Intervention (SCAI) has sought to standardize CS terminology to facilitate early-risk stratification, improve communication through defining clear shock strata, and expedite clinical decision-making [3]. The current paradigm classifies CS into five stages (A to E), with potential of progressing or improving between the stages [3]. Shock etiology also plays a role in CS recognition and progression. In AMI-CS, the abrupt loss of myocardial function coupled with inadequate circulatory compensation can lead to the rapid onset of severe shock [6]. In contrast, patients with chronic HF have adaptive mechanisms that compensate for their poor cardiac function, making the progression of HF-CS more gradual and less abrupt compared to AMI-CS [7]. Furthermore, isolated left ventricular (LV) failure, right ventricular (RV) failure, or biventricular failure complicate the phenotyping of this heterogeneous condition [8].

**Figure 1:**
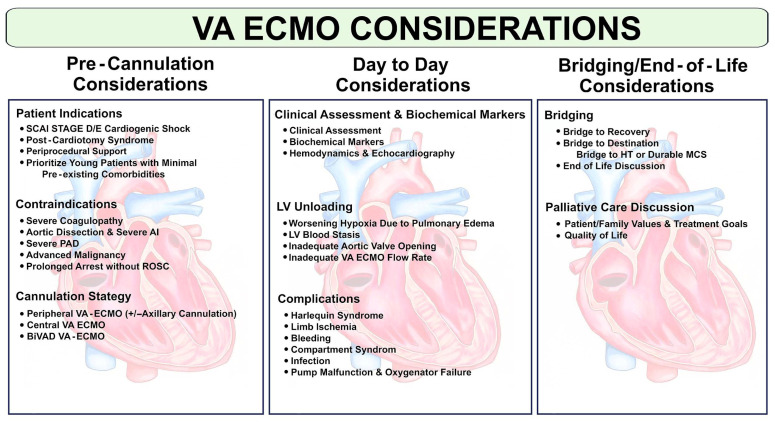
Central figure summarizing process, considerations, and approach to VA-ECMO support. Central figure summarizing important points in management prior to cannulization, while on VA-ECMO support, and while approaching cessation of support. Central Figure. AI = Aortic Insufficiency. BiVAD = Biventricular Assist Device. LV = Left Ventricle. HT = Heart Transplant. MCS = Mechanical Circulatory Support. PAD = Peripheral Arterial Disease. ROSC = Return of Spontaneous Circulation. SCAI = Society of Cardiovascular Angiography and Interventions.

Patients with advanced CS (i.e., SCAI stage D or E) can experience clinical deterioration despite being on intra-aortic balloon pump (IABP) and/or percutaneous left ventricular assist device (pLVAD) support [9]. While these devices may be beneficial in specific clinical scenarios of LV pump failure arising from AMI-CS and HF-CS, they are less effective at treating biventricular failure or complete cardiopulmonary collapse [10,11]. Consequently, VA-ECMO may have a role in managing the most advanced cases of CS [12]. VA-ECMO support can be instrumental in bridging patients to advanced heart failure (AHF) therapies or recovery. However, recent clinical trials have failed to show robust benefits of VA-ECMO as a definitive therapy for CS for these purposes [13,14,15,16,17,18,19]. This review aims to explore the appropriate indications for and optimal strategies in the use of VA-ECMO support in those with refractory CS (Figure 1).

## 2. Evidence for the Use of VA-ECMO

The 2025 American College of Cardiology/American Heart Association/American College of Emergency Physicians/National Association of Emergency Medical Services Physicians/Society for Cardiovascular Angiography and Interventions Guideline for the Management of Acute Coronary Syndromes has recently published guidance on the use of tMCSDs in the setting of AMI-CS [1]. Given the lack of mortality risk reduction observed in the IABP (intra-aortic balloon pump) SHOCK-II trial published in 2012 [2] and the ECLS-SHOCK trial published in 2023 [3], the guideline statement has given a class 3 recommendation, (level of evidence B) for routine use of either tMCSD strategy for those with AMI-CS. The use of microaxial intravascular flow pumps (Impella CP) in patients with STEMI with severe shock was given a class 2A recommendation (level of evidence B) following the publication of the contemporary, multicenter Danish–German Cardiogenic Shock (DanGer Shock) randomized trial in 2024 [19]. The trial investigated the risk of all-cause death at 180 days in patients with AMI-CS treated with an Impella CP compared to the standard of care. In a study cohort of 355 patients, there was a significant reduction in the risk of death at 180 days in the Impella CP arm (Hazard Ratio (HR) 0.74; 95% Confidence Interval (CI) 0.55–0.99, *p* = 0.04) compared to those with standard care. These trial results were in the setting of higher adverse event rates in the Impella CP arm (composite safety endpoint: severe bleeding, limb ischemia, hemolysis, device failure, aortic regurgitation [Relative Risk (RR) 4.74; [95% CI 2.36–9.55] and renal replacement therapy (RR 1.98, [95% CI 1.27–3.09]). Notably, patients with out-of-hospital cardiac arrest were excluded, contributing to a high rate of screen failures. Rates of bridging to AHF therapies were low and no significant difference was observed in 30-day mortality, which is a common endpoint for cardiogenic shock trials. Despite these limitations, it represents the first randomized controlled trial to demonstrate a mortality benefit with a tMCSD in CS care.

The EURO-SHOCK trial was the first multicenter randomized controlled trial of AMI-CS, designed to understand if early VA-ECMO use could improve outcomes in those with persistent CS following primary percutaneous coronary intervention (PCI) [4]. Patients were randomized to receive VA-ECMO or continue standard therapy post-PCI when persistent CS was present for at least 30 min. The trial was halted prematurely after randomizing 35 patients due to the impact of the COVID-19 pandemic. The 30-day all-cause mortality rates were 44% in the VA-ECMO group and 61% in the standard therapy group, with no statistically significant difference observed. Vascular and bleeding complications occurred more often in the VA-ECMO arm (21.4% vs. 0% and 35.7% vs. 5.6%, respectively).

Following EURO-SHOCK, the ECMO-CS trial randomized 117 patients with severe cardiogenic shock (SCAI Stage D or E), to immediate VA-ECMO or no immediate VA-ECMO in patients presenting with AMI-CS or HF-CS [5]. For the primary composite endpoint of death from any cause, resuscitated circulatory arrest, and implementation of additional mechanical circulatory support at 30 days, there was no significant difference between the immediate VA-ECMO group (63.8%) and the non-immediate VA-ECMO group (71.2%) (HR 0.72; [95% CI 0.46–1.12] *p* = 0.21). There were no significant differences in the risk of developing serious adverse events between groups.

The most contemporary trial of VA-ECMO use in CS was the Extracorporeal life support (ECLS) SHOCK multicenter, randomized study of patients with AMI-CS assigned to early ECLS plus usual medical treatment versus usual medical treatment alone [3]. Of the 417 patients included in the final analysis, roughly 50% of patients in both arms were classified as SCAI Stage C on presentation. For the primary outcome of all-cause mortality at 30 days, there was no significant difference in mortality between the VA-ECMO (47.8%) vs. no VA-ECMO groups (49%) (RR 0.98; [95% CI 0.80–1.19] *p* = 0.81). Bleeding and vascular complications occurred more frequently in the VA-ECMO arm (23.4% vs. 9.6%, RR 2.44; [95% CI, 1.50–3.95] and 11.0% vs. 3.8%, RR 2.86; [95% CI, 1.31–6.25], respectively). Data from this trial have informed the most recent 2025 ACC/AHA guidelines on the use of VA-ECMO in AMI-CS, where VA-ECMO was not recommended to be routinely used in AMI-CS. The differences in SCAI classifications between ECLS-SHOCK and ECMO-CS are notable, as the latter enrolled SCAI Stage D and above, while ECLS-SHOCK was comprised mostly SCAI Stage C patients. Furthermore, the high rate of cross-over in the ECMO-CS in an already a smaller trial may contribute to the null effect of the intervention. Though hard to execute these trials with uniform practice protocols, consistent indications for venting, cannulation timing in respect to revascularization, and the exclusion of AMI-CS cases of prolonged cardiac arrest, doing so may reduce patient heterogeneity and thus improve our understanding as to which patients may benefit from VA-ECMO in the setting of AMI-CS.

Reasons underpinning the lack of mortality benefit are plentiful. VA-ECMO-related complications likely outweighed the benefits of VA-ECMO and significantly increased short-term mortality risk. LV venting was underutilized (5.8% of patients) for a subset of patients who may have experienced ongoing significant congestion. Furthermore, many patients (77.7%) survived cardiac arrest prior to randomization. These patients may have had post-cardiac arrest stunning of the myocardium, leading to vasoplegia rather than CS, further heightening the risk of immediate mortality. It is less certain whether patients with SCAI Stage C and D without cardiac arrest may fare better with ECLS compared to other medical and device therapies. Following this guideline and recent large clinical trial, further large, randomized trials of VA-ECMO use in AMI-CS are not currently ongoing (Table 1).

## 3. Patient Selection

Trial data and practice guidelines do not suggest the routine use of VA-ECMO for either AMI-CS or HF-CS. Nonetheless, it still is a common tMCSD employed in advanced cases of CS, post-cardiotomy shock, primary graft failure after heart transplantation, and massive pulmonary embolism with hemodynamic collapse [19]. As already described, the most common candidate for VA-ECMO support would be those classified as SCAI D or E CS at VA-ECMO-capable centers [20,21]. Given the significant resource utilization needed for VA-ECMO deployment, including adequate staffing (cardiac surgeon or interventional cardiology, cardiac perfusion) and intensive care unit (ICU) and operating room availability, thought must be given to which patients may potentially benefit from this intervention.

The Survival after Venoarterial ECMO (SAVE) score is an actionable tool to identify pre-VA-ECMO factors associated with worse survival from refractory CS [22]. Patient-level data from 2003 to 2013 were extracted from the International Extracorporeal Life Support Organization registry, and of 3856 patients included in the registry, 1601 (42%) survived to discharge. Factors associated with worse lower rates of survival to discharge were chronic renal failure, multi-organ dysfunction, prolonged intubation ≥ 30 h, pre-VA-ECMO cardiac arrest, congenital heart disease, and low pulse pressure (<20 mmHg), with an overall model area under the curve (AUC) of 0.68. Single-center validation of this model has observed AUCs ranging from 0.7 to 0.9 [22,23]. s, When treating patients with AMI-CS where a rapid decision for escalation needs to be made, centers may consider implementing the following additional practical considerations as relative exclusion criteria for VA-ECMO: elderly patients > 75 years old, prolonged unwitnessed out-of-hospital cardiac arrest, concern for severe neurologic injury, severe comorbid conditions including dialysis dependence (for older patients), liver cirrhosis, severe chronic obstructive pulmonary disease with home-oxygen dependence, history of bleeding complications, excessive body mass index above 50 kg/m^2^, severe peripheral arterial disease, known advanced cancer, a “do not resuscitate” advanced directive, and unrepaired abdominal aortic aneurysm. This list is not exhaustive and represents some of the criteria centers may consider as relative contraindications. With the advent of electronic medical records, it may be possible to quickly discern if some of these conditions are present to better inform shock teams on the appropriateness of offering VA-ECMO in patients with AMI-CS.

Patients who are being considered for AHF therapies can be bridged with VA-ECMO, though the post-operative risk of both morbidity and short-term mortality is higher than in those not requiring this strategy. Based on the 2024 Society of Thoracic Surgeons Interagency Registry for Mechanically Assisted Circulatory Support (INTERMACS) report, patients with durable LVAD and critical CS requiring VA-ECMO (INTERMACS 1) had lower 1-year survival (80%) and 5-year survival (58%) compared to INTERMACS 4 patients, who do not require intravenous inotropes (1-year survival 87%, 5-year survival 63%) [24]. Furthermore, patients bridged to durable LVADs from VA-ECMO have a higher risk of right ventricular failure post-LVAD, with higher rates of temporary right ventricular assist device use [25,26], which is independently associated with a higher risk of 1-year mortality and LVAD-related adverse events. One-year survival outcomes for patients bridged to heart transplantation with VA-ECMO are lower than those of all-comers on the transplant waitlist (87% vs. 92%); overall outcomes have improved since the implementation of the 2018 allocation system and compared to the pre-policy listing system, where patients bridged with VA-ECMO had a 1-year survival estimated to be around 80%.

Device-related adverse events constitute an important consideration when implementing VA-ECMO support and invariably contribute to high rates of morbidity and mortality in patients with CS. Bleeding events, which can occur as local cannula-site events or as a systemic manifestation, are amongst the most common complications that occur post-VA-ECMO. Based on data from the Extracorporeal Life Support Organization (ELSO) registry on over 30,000 VA-ECMO runs, an overall bleeding complication rate was observed in 27.6% of patients over a course of follow-up of nearly 20 years from 2000 to 2019 [27]. Notably, reported bleeding rates in more recent cohorts are reported to be 15–18%. In the contemporary ECLS shock trial of patients with AMI-CS, rates of moderate or severe bleeding (overt bleeding with 3–5 g/dL drop requiring transfusion, intracranial hemorrhage, or severe fatal bleeding episode) occurred in 23.4% of those in the VA-ECMO arm compared to 9.6% in the control group [27]. Vascular complications necessitating intervention occurred in 11% of the VA-ECMO cohort. The types of cannulation and disease etiology may also affect bleeding risk and types of bleeding, whether intrathoracic, gastrointestinal tract, or cannula-related, in addition to the baseline coagulation profile and type and strength of anticoagulation regimen utilized.

Infection risk estimated post-VA-ECMO may be challenging to separate from the etiology of CS, type of cannulation, cardiac arrest at the time of cannulation, and presence of mechanical ventilation. Overall rates of infection necessitating treatment and not limited to bacteremia from a series of 488 patients with CS supported on VA-ECMO, published by Singh and colleagues [28], reported an infection rate of 35%. Bloodstream infections have a reported prevalence of 3% to 18% and 2.98 to 20.55 episodes per 1000 ECMO days in adults [21]. Risk of bacteremia may vary based on center-specific protocols, age of patient, days on support, and type of cannulation. Close attention to cannula access sites, and use of periprocedural antibiotics are recommended. Following cannulation, however, dosing of prophylactic antibiotics beyond the periprocedural time is not recommended, nor has it been demonstrated in any robust clinical study to have a treatment benefit.

Systemic anticoagulation can reduce the risk of thrombus formation within the cannulas and subsequently reduce the risk of thrombotic events. The risk of bleeding needs to be balanced with the use of anticoagulation, as the continuous flow of VA-ECMO support and use of the oxygenator can increase the risk of bleeding events due to acquired Von Willebrand disease, thrombocytopenia, and dilutional coagulopathies. A meta-analysis comprising nearly 1900 patients who required VA-ECMO for CS from Cheng and colleagues reported rates of neurologic adverse events, defined as intracerebral hemorrhage or ischemic stroke. Of the 630 patients adjudicated for neurologic complications, the cumulative pooled estimated rate of stroke was 5.9%.

Although VA-ECMO itself does not directly lead to acute kidney injury necessitating renal replacement therapy, the acute pathophysiologic insult of CS in patients ill enough to require VA-ECMO often coincides with severe end-organ function in the early phase of resuscitation, even if VA-ECMO restores systemic perfusion and improves left ventricular unloading. With varying definitions of acute kidney injury from the available literature, the reported incidence of acute kidney injury ranges from 33% to 56% post-VA-ECMO [20]. Rates of renal replacement therapy from Cheng and colleagues’ meta-analysis were 46% of roughly 1400 patients with available data [29]. Patients with pre-existing kidney dysfunction, advanced age, and diabetes, amongst several baseline characteristics, may place patients at even higher risk for kidney failure following VA-ECMO. These are important considerations when deciding candidacy for VA-ECMO in those with CS, as the presence of severe CKD or need for dialysis often will preclude patients from being candidates for AHF therapies such as LVAD, dual organ transplantation, or heart transplant alone. The possibility of safety net kidney transplant listing, in patients deemed non-recoverable on VA-ECMO, is feasible pending survival following the initial post-operative period. In this scenario, patients can receive high priority for a kidney transplant within the first year following a heart transplant [30].

## 4. VA-ECMO Cannulation

VA-ECMO management is a team effort among cardiothoracic surgery, cardiology, critical care, perfusion, specialized nursing, and palliative care teams [31,32]. Initiating VA-ECMO support requires a consensus among these specialists, based on clinical judgment and institutional resources. In most institutions, cardiothoracic surgery and heart failure cardiology serve as the main leaders in VA-ECMO management. As previously reported [12,21,31,32,33], VA-ECMO support involves five key phases: (1) activation, (2) cannulation, (3) ongoing management, (4) weaning, and (5) decannulation. In the following sections, we outline our approach to each phase.

### 4.1. Activation and Location

Following consensus, the procedural physician—either from cardiothoracic surgery or interventional cardiology—will activate the VA-ECMO team, which includes the operating room (OR) staff or the catheterization laboratory team and perfusionists [12,34]. Concurrently, critical care, emergency, or cardiology providers would coordinate appropriate bed placement in the intensive care units or paramedic transport [12]. More importantly, the location and cannulation strategy must be carefully determined to minimize complication risk associated with VA-ECMO support.

In many institutions, cardiothoracic surgery will place patients on VA-ECMO in the OR. This setting offers a controlled environment with immediate access to essential resources, including equipment, personnel, and space [34]. Familiarity is beneficial in the timely recognition and management of potential vascular complications, such as vena cava or right atrial perforation, which can be repaired expediently if in the OR. In this setting, given that cannulation is not performed in the intensive care unit, as much medical stabilization as possible should be attempted prior to transferring the patient to the OR for cannulation. For patients in external facilities, a direct transfer to the OR can be arranged to avoid additional delays when urgent cannulation is needed.

Cannulation at the bedside or in the catheterization lab is generally reserved for cases of critical urgency, such as ongoing advanced cardiac life support (ACLS) and/or hemodynamic instability [12]. Some institutions have developed protocols for out-of-hospital cannulation using specialized “mobile ECMO” units, though these require significant logistical and operational resources [35,36,37].

### 4.2. Cannulation Strategies

**Figure 2:**
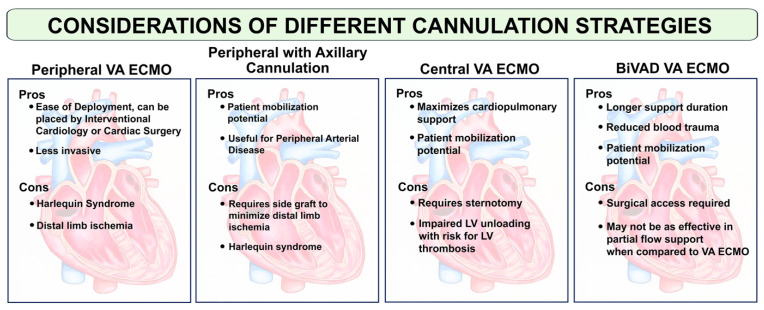
Considerations of various cannulation strategies. Peripheral VA-ECMO is the easiest to initiate but can be associated with poor distal limb perfusion. Peripheral VA-ECMO with axillary cannulation allows for patient mobilization but requires a side graft and may provide lower flows. Central VA-ECMO requires a sternotomy but provides the most flow among the VA-ECMO options. BiVAD VA-ECMO also requires surgical placement, but can provide longer, more durable support. BiVAD = Biventricular Assist Device. LV = Left Ventricle.

Cannulation techniques and necessary equipment have been well documented in the literature [32,38,39]. These approaches are influenced by the operator’s expertise, institutional resources, and clinical preferences. In brief, VA-ECMO cannulation involves the placement of a large-bore, inflow (venous) cannula for deoxygenated blood drainage and an outflow (arterial) cannula for oxygenated blood return [31]. These cannulas are connected to a centrifugal pump, which can provide flow rates of up to 7–8 L/min, along with an oxygenator to facilitate gas exchange [31]. Here, we highlight critical considerations and potential pitfalls associated with VA-ECMO cannulation (Figure 2).

**Central VA-ECMO:** The insertion site for the outflow cannula determines whether VA-ECMO is classified as “central” (ascending aorta) or “peripheral” (femoral or axillary artery). The choice between central and peripheral cannulation depends on the clinical situation as well as the advantages and disadvantages of each technique.

Central VA-ECMO cannulation is performed in the OR via median sternotomy. The arterial cannula is inserted into the ascending aorta through a 10 mm Hemashield graft (Maquet, Rastatt, Germany) sewn onto the aorta. To improve stability, the graft–cannula apparatus is tunneled to a subxiphoid position [32]. The venous cannula is placed in the common femoral vein, terminating at the right atrium. Cannula sizes are determined by body size to optimize VA-ECMO flow. If LV venting is indicated, a large-bore cannula may be secured to the right pulmonary vein–left atrium junction, and would terminate either in the left atrium or ventricle. This left atrial cannula can be connected to the venous (right atrial) cannula in a “Y” configuration to facilitate maximal blood drainage into the circuit [32,40,41]. Another configuration can utilize biventricular mechanical circulatory support (MCS), which entails connecting an outflow venous cannula to the pulmonary artery through a graft/cannula system, and then that blood volume would be diverted from a left atrial cannula into the ascending aorta [32].

These approaches offer several advantages, including the ability to achieve high flow rates and to provide full cardiopulmonary support [32]. Central VA-ECMO support ensures adequate perfusion of all vital organs and allows for patient mobilization, thus minimizing the risk of deconditioning. However, there are inherent risks, such as sternotomy complications, mediastinal bleeding and infection, and impaired LV unloading (when a vent is not used), leading to worsening congestion in cases where patients have a significant reliance on the ECMO circuit for circulatory support.

**Peripheral VA-ECMO:** This cannulation strategy presents several advantages of being less invasive, having easier deployment (particularly in emergency situations), and avoiding sternotomy [12,33]. There also may be fewer bleeding complications given the less invasive approach. There are disadvantages worth noting. “North–South syndrome” (described below), increased LV afterload leading to ventricular distention and thrombosis formation in the LV and aortic root, and the potential for distal limb ischemia [42] must be monitored following peripheral cannulation.

Peripheral VA-ECMO can also be performed via a surgical cutdown in the OR. The venous cannula is placed in the same manner as central VA-ECMO. A 10 mm Hemashield graft is sewn to the ipsilateral or contralateral femoral artery (depending on the body habitus), through which the arterial cannula is inserted. This technique eliminates the need for a distal perfusion catheter to prevent lower extremity ischemia. A disadvantage of other forms of peripheral cannulation via axillary artery is upper extremity hyperemia, ischemia, and lower flows due to smaller vessel caliber. In scenarios requiring more complete venous drainage, an additional cannula may be placed via the internal jugular vein, terminating in the pulmonary artery.

Peripheral VA-ECMO cannulation can be performed percutaneously at bedside or in the catheterization lab. In these settings, equipment must be stored in a mobile cart for easy access. A distal perfusion catheter should be considered when placing the arterial cannula to minimize the risk of limb ischemia [42].

**ECMO Pump:** Following cannulation and connecting to the ECMO circuit, the flow rate is gradually increased to 4–6 L/min, which corresponds to a cardiac index of approximately 2.2 L/min/m^2^. The Centrimag (Thoratec, Pleasanton, California) centrifugal pump has the advantages of durability, convenience, and decreased tendency toward hemolysis [43]. Other centrifugal pumps, such as the CardioHelp (Maquet, Rastatt, Germany) and TandemHeart (TandemLife, Pittsburgh, Pennsylvania) systems, may also be utilized. The CardioHelp system has the advantage of being compact, thus facilitating intra-facility transport [44].

**Determining which cannulation strategy to deploy:** The decision to deploy central versus peripheral VA-ECMO is individualized and based on clinical presentation, weighing the benefits and risks associated with each approach [21,31,33]. Our center frequently uses central VA-ECMO as the support strategy for refractory CS resulting from acute-on-chronic heart failure and large myocardial infarction. This approach maximizes cardiopulmonary support because it is not limited by cannula length or size, thus ensuring adequate perfusion to assist end-organ recovery. Patients may be extubated and mobile for rehabilitation. If a “Y” cannula (left atrial cannula connected to venous cannula) is used, the right atrial cannula can eventually be removed, creating a temporary LV assist device (LVAD) configuration which serves as a trial for durable LVAD [45]. In this scenario, we would aim for the redo sternotomy to be within 1–2 weeks to minimize complications.

Peripheral VA-ECMO is best considered for those who are too unstable to be mobilized or who are experiencing ongoing ACLS, either at bedside or in the catheterization lab. This strategy may also be preferred in patients for whom multiple sternotomies should be avoided. Regardless, multidisciplinary-based decisions are needed to choose the best cannulation strategy to optimize outcomes and to minimize risks.

## 5. Left Ventricular Unloading and Venting

**Figure 3:**
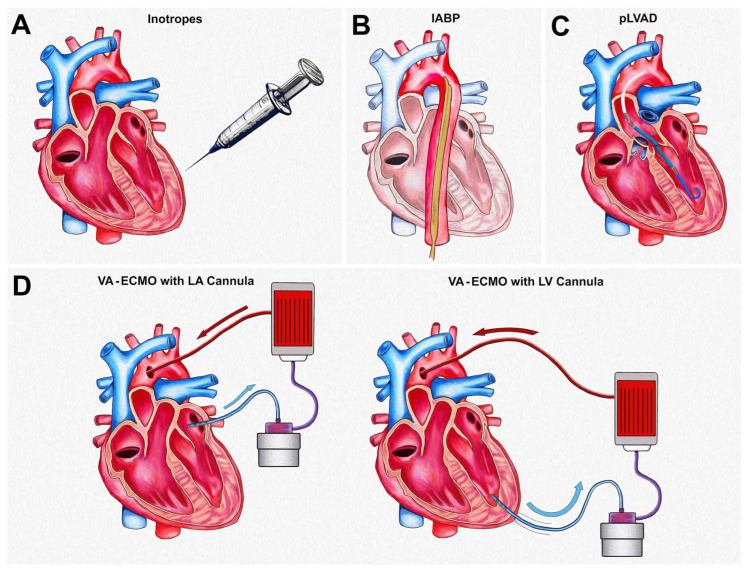
Left ventricular unloading and venting methods. (**A**) Inotropes can be considered for patients requiring minimal unloading or low ECMO flows, though they increase myocardial oxygen demand and can increase arrhythmia burden. (**B**) An intra-aortic balloon pump (IABP) is also beneficial in patients requiring less support and is most easily placed among MCS options, though may result in inadequate venting on full ECMO support. (**C**) Percutaneous left ventricular assist devices (pLVADs) result in a higher degree of LV unloading and venting, and should be considered for patients in profound shock with significantly elevated LV pressures. (**D**) Surgical left atrial (LA) or left ventricular (LV) apical cannulation is more invasive, but provides maximal flow and direct LV unloading; it can be considered for patients in whom peripheral VA-ECMO is not feasible due to peripheral arterial disease.

The retrograde flow from the VA-ECMO circuit generates afterload pressure against the LV, potentially increasing myocardial workload, impairing LV energetics, and exacerbating LV dysfunction [46,47]. This process can result in LV distention, pulmonary venous congestion, and impaired aortic valve function, leading to blood stasis and mitral valve regurgitation. LV unloading refers to strategies that reduce myocardial workload and promote recovery, whereas LV venting alleviates the congestive sequela of increased afterload. Several approaches have been proposed to achieve these goals (Figure 3) [46].

Inotropic and IABP support may be sufficient for LV venting. However, there is a theoretical risk of inadequate LV unloading, which may not support LV recovery [46]. The pLVAD has been suggested to achieve both unloading and venting. A left atrial cannula via surgical placement (“Y” to the venous cannula) or through a percutaneous atrial septostomy is an alternative [48,49,50]. Adding a second venous cannula in the pulmonary artery could also be considered for venting. Observational studies have suggested potential hemodynamic advantages [51,52], and some prospective studies have shown potential benefits in using pLVAD venting when compared to those on VA-ECMO alone [53,54]. A recent single-center study (PAPO-Flow Study) demonstrated that following VA-ECMO flow and afterload increase, the rise in LV filling pressure was counterbalanced by RV preload decrease, resulting in neutral effects for most patients with minimal rise in pulmonary capillary wedge pressure (PCWP) [55]. Based on this contemporary study, it is plausible that patients with some degree of intrinsic pulsatility may represent a cohort where an additional device or therapy to promote LV unloading may not always be required. Nevertheless, there is limited evidence from randomized clinical trials to guide clinical decision-making [56]. Several trials are ongoing to assess various LV venting strategies in patients requiring VA-ECMO [47,57]. The EARLY-UNLOAD trial did not observe a survival benefit with the use of routine early unloading via percutaneous transseptal left atrial cannulation in patients undergoing VA-ECMO, though a significant rate of cross-over in the conventional group, exceeding 50% of the control arm, may have had a confounding effect in this smaller clinical trial. Ultimately, it is advised to follow practical markers of left ventricular ejection during VA-ECMO support, such as aortic pulsatility, the use of a Swan-Ganz catheter to assess pulmonary artery pressures, and an echocardiogram to ensure no blood stasis in the left ventricle prior to introducing mechanical left ventricular unloading, as patient-specific physiology may simply guide the need for these additional interventions.

**Practical considerations:** Balancing the goals of LV recovery or bridge to advanced HF therapies while minimizing adverse events drives the decision for various cannulation strategies and LV venting decisions. After cannulation, regular reassessment is essential. Indicators for escalation to mechanical LV venting include worsening hypoxia due to pulmonary edema; LV blood stasis; and inadequate aortic valve opening [46,47]. More importantly, the VA-ECMO flow rate should be titrated to maintain cardiac index > 2.2 L/min/m^2^ and reduce LV filling pressures. The primary objective of this goal is to optimize coronary and RV perfusion and to improve myocardial workload in addition to end-organ recovery. Therefore, LV venting may be necessary when the goal of achieving an adequate VA-ECMO flow rate is compromised.

If LV venting is deemed necessary, our center may pivot to a strategy of central VA-ECMO with a left atrial (or additional pulmonary artery) cannula to provide optimal cardiopulmonary support and to enhance patient mobility. In certain cases, the venous (right atrial) cannula may be removed to convert to an LVAD configuration, as described above. Furthermore, the femoral venous cannula may be transitioned to the internal jugular vein to improve mobility. For peripheral VA-ECMO support, LV venting may not be needed if the residual LV function is acceptable, though this configuration may be less likely to promote adequate myocardial recovery. In such cases, the use of an IABP or pLVAD (Impella CP or 5.5) for short-term treatment (<1 week of support) are potential options to allow for adequate LV unloading.

## 6. Comprehensive Daily Considerations

Vigilance and multidisciplinary care are foundational to optimizing outcomes and mitigating complications. In this context, we have outlined our daily approach to VA-ECMO care.

**Circuit components:** The circuit consists of pressure and oxygen saturation monitors, air bubble detectors, temperature and flow sensors, hemoconcentrators, and safety alarms [58]. The oxygenator has a gas-permeable polymer membrane to facilitate gas exchange, with sweep rate adjustments to control carbon dioxide removal. A heat exchanger maintains blood temperature at 37 °C to enhance gas exchange efficiency.

### 6.1. Routine Assessment

Proactive monitoring facilitates the early recognition and expectant management of potential complications. Evaluation should include inspection of cannulation sites for bleeding, infection, and positioning/migration; assessment for distal limb ischemia in peripheral VA-ECMO; and surgical wound integrity [59]. Circuit surveillance entails monitoring for thrombus formation and oxygenator performance [59]. Laboratory tests monitor renal and hepatic function, urine output, lactate clearance, coagulopathy, and blood gases. Hemodynamic data from arterial lines and pulmonary artery catheters should be utilized to assess treatment response [60].

Hourly checks of circuit flow, pressures, and oxygenator function should be performed, as well as documenting cannula depth and position to detect cannula migration. Line pressures should be monitored at least daily. The pre- and post-membrane pressure represents the driving pressure from the pump through the circuit. Pre-membrane pressure should ideally be less than 300 mmHg. The membrane gradient (delta p) should be 10–40 mmHg. Elevated line pressure may represent inadequate cannula size and ongoing suctioning. Additionally, elevated delta p could signal thrombus formation within the oxygenator which should be monitored for ona. Routine basis. Rising transmembrane gradients or worsening gas exchange may indicate a need for oxygenator replacement.

Echocardiography (both transthoracic and intra-operative transesophageal) is essential at baseline, during clinical changes, and when performing ramp studies [61,62]. Key parameters to assess are the degree of LV distension, thrombus burden, mitral regurgitation severity, aortic valve opening, and RV function. These findings inform decisions on LV venting strategies, particularly in cases of pulmonary edema or limited aortic valve opening.

### 6.2. Hemolysis, Coagulopathy, and Anticoagulation

Bleeding and coagulopathy are multifactorial, arising from systemic anticoagulation, inflammatory activation, and clotting factor disruption [63,64]. Platelet trends and thromboelastography can be used to guide transfusion and anticoagulation decisions [64,65]. Hemolysis is common, and the accompanying elevated plasma-free hemoglobin contributes to vasoconstriction, endothelial dysfunction, platelet aggregation, and tissue hypoxia, thus feeding into the cycle of coagulopathy [66,67]. To address hemolysis, optimizing cannula size and flow rates would reduce shear stress [68]. Judicious anticoagulation with unfractionated heparin or bivalirudin is most often used to avoid thrombus formation, with close attention to bleeding related to the ECMO circuit [65,69,70]. In the case of hemolysis or visible intracardiac thrombus, a stronger anticoagulation protocol may be considered by the treatment team.

### 6.3. Pulmonary, Ventilation, Anemia, and Oxygen Delivery

In CS, hypoxia related to pulmonary infiltrates should be resolved quickly once on VA-ECMO support. Best practices for mechanical ventilation while on ECMO support for respiratory failure are translatable to patients with CS [71,72]. In general, optimizing oxygenation and ventilation requires balancing the ventilator and VA-ECMO support for the recovering lung parenchyma. Systemic arterial oxygen saturation should be ≥92% while avoiding hyperoxia and hypercarbia [73]. Positive pressure ventilation may reduce alveolar edema and improve alveolar recruitment. Minute ventilation could supplement sweep rate to manage hypercarbia. Similarly to other centers [74], we favor early extubation (“awake ECMO” approach) to reduce barotrauma risk, support pulmonary recovery, and enhance physical mobilization [75,76]. Whenever feasible, we optimize the circuit to maintain spontaneous ventilation and minimize the need for reintubation.

Furthermore, anemia from hemolysis, phlebotomy, hemorrhagic complications, and hemodilution impairs oxygen delivery, flow rate, and circuit efficacy [77,78]. A hemoglobin target of 7–8 g/dL strikes a balance between oxygenation needs and transfusion risk [79,80,81]. Higher thresholds (>10 g/dL) may increase transfusion requirements and mortality [80,81].

### 6.4. North–South Syndrome (Harlequin Syndrome)

Commonly seen in peripheral cannulation, cerebral and upper body hypoxia is due to abnormal pulmonary mechanics and maldistribution of oxygenated blood [31,82]. The phenomenon occurs when the junction (i.e., mixing cloud) of the oxygenated retrograde blood flow and poorly oxygenated anterograde blood is distal to the transverse aorta. Diagnosis involves identifying discrepancies between arterial blood gases from the right upper extremity and the oxygen saturation from the left [63]. Management includes increasing circuit flow, conversion to central VA-ECMO, or adding an additional venous return cannula to oxygenate blood entering the pulmonary circulation [83].

### 6.5. Multidisciplinary Collaboration and Communication

Daily multidisciplinary rounds involving intensivists, cardiologists, perfusionists, nurses, therapists, and pharmacists facilitate cohesive care [84]. Clear communication with patients and families aids in shared decision-making and aligning goals of care. This section highlights several care teams that are also integral to success.

**Infection Control**: Daily evaluation for bacteremia, cannula site infections, and oxygenator contamination is essential [85,86]. Cultures should be obtained promptly when infection is suspected. Empiric antimicrobial coverage should be balanced with stewardship principles to minimize antibiotic resistance.

**Neurologic and Delirium Surveillance:** Neurologic monitoring is a central component, especially in awake ECMO protocols [74,86,87]. Daily assessments should screen for stroke, seizures, and hypoxic–ischemic injury. Delirium should be evaluated using validated tools such as CAM-ICU and RASS scores [87]. Sedation should be minimized to promote cognitive recovery and reduce critical-illness myopathy [87].

**Pharmacy:** VA-ECMO alters pharmacokinetics due to circuit sequestration and organ dysfunction [88,89]. Pharmacy ensures appropriate drug selection and dosing. They also conduct therapeutic drug monitoring for antimicrobials, sedatives, and anticoagulants.

**Psychological Support:** Addressing anxiety, depression, and post-traumatic stress disorder is important, especially in awake VA-ECMO patients. Family support and counseling are essential during prolonged or uncertain courses. The palliative care and psychology teams help with coping mechanisms for both patient and family [90,91].

**Rehabilitation and Nutritional Support:** Early mobilization improves intensive care unit survival and is beneficial to patients who are on “awake VA-ECMO support” [92,93]. Both physical and occupational therapy should begin early and be tailored to patient stability and cannula configuration. Speech therapy may be needed to assist with communication and swallowing following extubation.

Early introduction of enteral nutrition helps preserve gut integrity and may reduce mortality [94,95]. Nutritional support should be individualized to meet the elevated demands of the ongoing catabolism in critical illness, with attention to both caloric and micronutrient intake [96,97]. Glycemic control may also help to prevent complications while on VA-ECMO support [98]. Additionally, opioid use should be judiciously balanced between pain control and risk of constipation and opioid-induced ileus.

## 7. Weaning VA-ECMO

**Figure 4:**
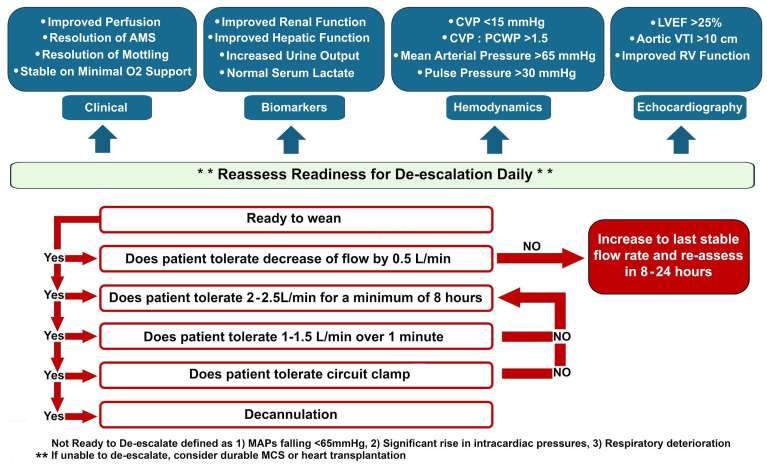
Suggested approach to weaning to VA-ECMO support. Common pitfalls to weaning include (1) weaning too early; improvement across multiple clinical endpoints and stable hemodynamics are prerequisites to weaning consideration, (2) weaning too quickly; flows should be decreased by 0.5 L/min at a time and tolerance assessed prior to further weaning, (3) decannulation prior to assessment on minimal flows; patients need to tolerate 2–2.5 L/min for at least 8 h and 1–1.5 L/min for a minute prior to safe decannulation, and (4) insufficient clinical assessment throughout wean; PA catheter guided hemodynamics, echocardiographic parameters, mental status, renal and liver function, and pulmonary function all need to be synthesized throughout the wean. AMS = Altered Mental Status, CVP = Central Venous Pressure, LVEF = Left Ventricular Ejection Fraction, MAP = Mean Arterial Pressure, MCS = Mechanical Circulatory Support. PCWP = Pulmonary Capillary Wedge Pressure, RV = Right Ventricle, VTI = Velocity Time Integral.

Though no validated VA-ECMO weaning strategies exist, the fundamental principle is that once end-organ recovery is realized, improvement in systolic function is evaluated to determine the need for VA-ECMO support while balancing with the competing risk of long-term survival being at least one year on optimal medical therapy [99]. This dynamic risk assessment is multidisciplinary and completed daily. If a patient cannot be liberated from VA-ECMO support, then the flow rate should be reduced to the lowest tolerable level to mitigate complication risk [100]. Herein, we discuss the approach at our institution to liberate patients from VA-ECMO support (Figure 4).

Weaning readiness is determined by clinical, laboratory, hemodynamic, and echocardiographic assessments. Clinical markers include improved mentation and successful extubation onto minimal oxygen support. Laboratory markers should point toward normalizing renal and hepatic function, as well as increasing urine output and normal serum lactate levels. In patients with severe renal injury, renal replacement therapy may be needed to manage uremia and pulmonary edema. Respiratory status should be stable on minimal oxygenator support (~0.5–1 L/min sweep, FiO_2_ 100%); lower FiO_2_ would induce a right-to-left shunt and cyanosis. On hemodynamic assessment, benchmark values include a central venous pressure (CVP) goal < 15 mmHg, CVP-PCWP ratio > 1.5, mean arterial pressure > 65 mmHg, and pulse pressure > 20–30 mmHg. Echocardiographic parameters should include improving LV systolic function (ideally LV ejection fraction > 25%), robust aortic valve opening during cardiac cycles, and absence of LV and RV distension and blood stasis [101]. Patients should be preferably be on minimal inotropic and pressor support.

Our standard approach begins by reducing the Centrimag^TM^ pump speed by 100–200 RPM daily (equating to a 0.2–0.5 LPM decrease in flow rate). Once the support level falls below 3.0 LPM, a comprehensive reassessment is performed. If the patient remains stable, an echocardiography-guided ramp study is conducted at approximately 2.0 LPM, followed by an evaluation at less than 0.1 LPM (“clamp”) to assess myocardial geometry and function. Final readiness is determined in a multidisciplinary discussion. Decannulation is performed at bedside, followed by ligation and removal of the grafts under monitored anesthesia care (MAC) or general anesthesia in the operating room for patients on central VA-ECMO support or with peripheral VA-ECMO with a graft on the femoral artery. Following decannulation, we would continue optimizing CVP goal < 15 mmHg and cardiac index > 2.2 L/min/m^2^, and eventually, transition from inotropic support to guideline-directed medical therapy.

## 8. Bridging to AHF Therapies

Patients placed on VA-ECMO for refractory CS should be considered for AHF therapies, including heart transplantation and durable LVAD, in the absence of absolute contraindications. If hemodynamics data are available, requirements include cardiac index < 1.8 L/min/m^2^ or <2.0 L/min/m^2^ on inotropes, PCWP > 15 mmHg, and systolic blood pressure < 90 mmHg within 24 h or any of the following within a 24 h period. If hemodynamics data are not available, any of the alternative criteria can be applied if occurring within 24 h prior to VA-ECMO: cardiopulmonary resuscitation, arterial lactate > 4 mmol/L, systolic blood pressure < 70 mmHg, and liver function tests (aspartate transaminase or alanine transaminase) > 1000 U/L. Patients on VA-ECMO can be listed at the highest priority for heart transplantation (UNOS Status 1), and will need re-justification at 1 week following initial listing showing an inability to wean from VA-ECMO with a documented contraindication for a durable LVAD [102].

One year post-transplant survival initially following this policy implementation in 2018 was lower than other candidates at 75%, though improvements in overall survival have been observed in more recent audits of UNOS data, now approaching 85% at 1 year [28]. A durable LVAD should also be considered for a patient with refractory CS on VA-ECMO and is similarly associated with higher clinical risk, specifically mortality, RV failure necessitating a temporary RV assist device, and dialysis, compared to patients who do not require as high an amount of circulatory support [26]. Patients with critical CS requiring VA-ECMO, based on the most recent INTERMACS report with the contemporary HeartMate 3 LVAD, have a lower overall one-year survival of 80%, compared to 88% for patients supported on inotropic therapy alone [24].

## 9. Future Directions

Patient selection, resource allocation, and ethical considerations remain central topics in optimizing VA-ECMO support. Decision-making should be individualized given the limited high-quality evidence and the knowledge gap in HF-CS. While recent trials have primarily focused on MI-CS, early VA-ECMO escalation does not appear to have a survival benefit compared to an initial conservative approach [14]. Further research is needed to refine diagnostic and therapeutic strategies tailored to HF-CS patients. Another unresolved issue is the role of LV venting in VA-ECMO management. Identifying those needing mechanical LV venting and/or unloading is unclear. Non-tMCSD methods could be explored as potential alternatives [103].

Prolonged immobilization and VA-ECMO support are associated with critical-illness myopathy and polyneuropathy. Incorporating pre-habilitation strategies, particularly for patients being bridged to AHF therapies, could speed up postoperative recovery [104]. However, current mobility interventions pose logistical challenges. Bed bikes and standing hospital beds may cause excessive strain on femoral access sites [105]. Upper body cannulation can facilitate movement but may not provide adequate circulatory support. Emerging modular VA-ECMO systems, such as “ECMO TO GO”, offer compact designs to complement patient freedom [106]. Future studies would need to balance adequate circulatory support with functional independence.

Beyond technical considerations, ethical dilemmas could complicate VA-ECMO therapy. One concern is VA-ECMO support as a “bridge to nowhere”, in which life is prolonged without meaningful recovery. Having a defined “landing zone” following VA-ECMO initiation is essential to prevent medically futile interventions. Discontinuing VA-ECMO support in non-recovering patients raises ethical challenges when the perspectives of the family, patient, and clinical team are misaligned. Both family and clinical providers consequently may experience distress and uncertainty in navigating these complex scenarios. Structured ethical frameworks, including communication and shared decision-making guidelines from the institution, would improve patient and provider satisfaction [107]. Developing formal selection criteria, LV venting strategies, and earlier transition to AHF therapies could improve outcomes and minimize complications. Integrating artificial intelligence to create adaptive models, rather than current static prediction tools, could be employed to help institutions allocate resources more efficiently, prioritizing those most likely to benefit [108]. Ultimately, each center that has a VA-ECMO program should conduct regular reviews of protocols and selection criteria, with critical revision for transparency within the hospital system and to support ongoing efforts in quality improvement and furthering the understanding of patient criteria and practices that are associated with the best outcomes in what is a highly morbid clinical scenario. Uniform, firm criteria in evaluating candidacy VA-ECMO may be ethically impermissible. A holistic framework including survival probability along with patient and center preference needs to be incorporated when constructing a VA-ECMO program.

## 10. Conclusions

“*If you want to go fast, go alone. If you want to go far, go together*.”

African proverb—[109]

Although current evidence suggests limited benefits of VA-ECMO support in refractory cardiogenic shock, it remains the most effective cardiopulmonary support system. Because of significant heterogeneity, management is highly individualized, as no universal strategy exists. A multidisciplinary effort is crucial to minimize complications and optimize outcomes. Ultimately, the goal would be achieving the holy grail (a one-year survival rate of ≥80% with good quality of life), underscoring the importance of refining patient selection, timing of initiation, meticulous management, and early transition to AHF therapies in those needing VA-ECMO support.

## Figures and Tables

**Table 1:** Trials and major observations in VA-ECMO.

Study	Study Type	Population and Study Groups	Results	Complications
Brunner et al. (2019) [13]	Randomized trial	AMI-CS VA-ECMO (*n* = 21) vs. Standard of care (*n* = 21)	**1° Endpoint:** 30-day LVEF 50.0% in VA-ECMO vs. 50.8% in standard of care (*p* = 0.86) **Conclusion**: No change in LVEF at 30 days	Stroke (5% vs. 5%, *p* = 0.10) Vascular complications (10% vs. 0%, *p* = 0.49) Life-threatening bleeding (19% vs. 14%, *p* = 0.10) Sepsis (43% vs. 33%, *p* = 0.75) Reinfarction (10% vs. 5%, *p* = 0.10) Stent thrombosis (10% vs. 5%, *p* = 0.10)
Ostadal et al. (2023) [14]	Randomized trial	Rapidly deteriorating or severe CS Immediate VA-ECMO (*n* = 58) vs. Early Conservative (*n* = 59) **CS Cause**: AMI-CS (62%) and HF-CS (23%), MI complications (2.6%)	**1° Endpoint:** 30-day composite of all-cause death, resuscitated circulatory arrest, and another tMCSD 63.8% in Immediate VA-ECMO vs. 71.2% in Early Conservative (HR 0.72 [95% CI 0.46–1.12], *p* = 0.21) 39% Early Conservative: Cross-over to VA-ECMO support; Primarily SCAI Stage D and E **Conclusion**: No difference in rates of death and other key endpoints at 30 days	Serious adverse events similar (60.3% vs. 61.0%)
Thiele et al. (2023) [15]	Randomized trial	AMI-CS VA-ECMO (*n* = 209) vs. Standard of care (*n* = 208)	**1° Endpoint:** 30-day all-cause death: 47.8% in VA-ECMO group vs. 49.0% in control (RR 0.98 [95% CI 0.80–1.19] *p* = 0.81) 12.5% control cross-over to VA-ECMO support; Primarily SCAI Stage C **Conclusion**: No difference in rates of death at 30 days	Moderate or severe bleeding: 23.4% vs. 9.6%, RR: 2.44 (1.50–3.95) Vascular complications needing intervention 11% vs. 3.8%, RR 2.86 (1.31–6.25)
Kim et al. (2023) [16]	Randomized trial	CS receiving VA-ECMO Early LV unloading with trans-septal LA cannulation (*n* = 58) vs. Conventional VA-ECMO (*n* = 58) **CS Cause**: AMI-CS (66%), HF-CS (13.8%), myocarditis (8.6%), others (11.2%)	**1° Endpoint:** 30-day all-cause death: 46.6% in early LV unloading vs. 44.8% in conventional (HR 1.02 [95% CI 0.59–1.74] *p* = 0.94) 50% Conventional: Cross-over to trans-septal LA cannulation; Primarily SCAI Stage D **Conclusion**: No difference in rates of death at 30 days	Cardiac tamponade (*n* = 2) needing pericardiocentesis in Early LV unloading No aorta injury Iatrogenic ASD (*n* = 3) post-procedure in Early LV unloading
Banning et al. (2023) [17]	Randomized trial	AMI-CS VA-ECMO (*n* = 17) vs. Standard of care (*n* = 18)	**1° Endpoint:** 30-day all-cause death: 43.8% in VA-ECMO vs. 61.1% in standard of care (HR 0.56 [95% CI 0.21–1.45] *p* = 0.22) **Conclusion**: Incomplete trial due to limited enrollment to study the effect of VA-ECMO post-PCI in patients with severe CS	Vascular and bleeding complications (21.4% vs. 0% and 35.7% vs. 5.6%, respectively)

AMI-CS = acute myocardial infarction-related cardiogenic shock. ASD = atrial septal defect. HF-CS = heart failure-related cardiogenic shock. HR = hazard ratio. LA = left atria. LVEF = left ventricular ejection fraction. RR = relative risk. tMCSD = temporary mechanical circulatory support device (other than VA-ECMO). VA-ECMO = venoarterial extracorporeal membrane oxygenation.

## Data Availability

No original data source was used in the contemporary review.

## References

[1] Feinman J., Tomey M.I., Palazzolo M.G., Martillo M., Ronquillo M., Moss N., Serrao G., Bohula E.A., Berg D.D., VAN Diepen S. (2025). Differences Between Ischemic and Nonischemic Cardiomyopathy in Heart Failure Related Cardiogenic Shock. J. Card. Fail..

[2] Bertaina M., Morici N., Frea S., Garatti L., Briani M., Sorini C., Villanova L., Corrada E., Sacco A., Moltrasio M. (2023). Differences between cardiogenic shock related to acute decompensated heart failure and acute myocardial infarction. ESC Heart Fail..

[3] Brener M.I., Rosenblum H.R., Burkhoff D. (2020). Pathophysiology and Advanced Hemodynamic Assessment of Cardiogenic Shock. Methodist DeBakey Cardiovasc. J..

[4] Hernandez-Montfort J., John K.J., Goldstein D., Lorusso R., Sinha S.S., Goodman R., Natov P., Li S., Li B., Kanwar M. (2025). Clinical outcomes of cardiogenic shock patients supported with VA-ECMO: Insights from the Cardiogenic Shock Working Group. J. Heart Lung Transplant..

[5] Mariani S., Napp L.C., Coco V.L., Delnoij T.S., Luermans J.G., ter Bekke R.M., Timmermans C., Li T., Dogan G., Schmitto J.D. (2020). Mechanical circulatory support for life-threatening arrhythmia: A systematic review. Int. J. Cardiol..

[6] Dhakam S., Khalid L. (2008). A Review of Cardiogenic Shock in Acute Myocardial Infarction. Curr. Cardiol. Rev..

[7] Sinha S.S., Rosner C.M., Tehrani B.N., Maini A., Truesdell A.G., Ben Lee S., Bagchi P., Cameron J., Damluji A.A., Desai M. (2022). Cardiogenic Shock from Heart Failure Versus Acute Myocardial Infarction: Clinical Characteristics, Hospital Course, and 1-Year Outcomes. Circ. Heart Fail..

[8] Kuchibhotla S., Esposito M.L., Breton C., Pedicini R., Mullin A., O’KElly R., Anderson M., Morris D.L., Batsides G., Ramzy D. (2017). Acute Biventricular Mechanical Circulatory Support for Cardiogenic Shock. J. Am. Heart Assoc..

[9] Hill K.L., Rustin M.A., Asche M.A., Bennett C.E., Patel P.C., Jentzer J.C. (2023). Cardiogenic Shock Classification and Associated Mortality Risk. Mayo Clin. Proc..

[10] Kar B., Basra S.S., Shah N.R., Loyalka P. (2012). Percutaneous Circulatory Support in Cardiogenic Shock. Circulation.

[11] Kalapurakal G., Chau V.Q., Imamura T., Tolia S., Sciamanna C., Macaluso G.P., Joshi A., Pillarella J., Pauwaa S., Dia M. (2024). Haemodynamic effects of intra-aortic balloon pumps stratified by baseline pulmonary artery pulsatility index. ESC Heart Fail..

[12] Rao P., Khalpey Z., Smith R., Burkhoff D., Kociol R.D. (2018). Venoarterial Extracorporeal Membrane Oxygenation for Cardiogenic Shock and Cardiac Arrest. Circ. Heart Fail..

[13] Brunner S., Guenther S.P., Lackermair K., Peterss S., Orban M., Boulesteix A.-L., Michel S., Hausleiter J., Massberg S., Hagl C. (2019). Extracorporeal Life Support in Cardiogenic Shock Complicating Acute Myocardial Infarction. J. Am. Coll. Cardiol..

[14] Ostadal P., Rokyta R., Karasek J., Kruger A., Vondrakova D., Janotka M., Naar J., Smalcova J., Hubatova M., Hromadka M. (2023). Extracorporeal Membrane Oxygenation in the Therapy of Cardiogenic Shock: Results of the ECMO-CS Randomized Clinical Trial. Circulation.

[15] Thiele H., Zeymer U., Akin I., Behnes M., Rassaf T., Mahabadi A.A., Lehmann R., Eitel I., Graf T., Seidler T. (2023). Extracorporeal Life Support in Infarct-Related Cardiogenic Shock. N. Engl. J. Med..

[16] Kim M.C., Lim Y., Lee S.H., Shin Y., Ahn J.H., Hyun D.Y., Cho K.H., Sim D.S., Hong Y.J., Kim J.H. (2023). Early Left Ventricular Unloading or Conventional Approach After Venoarterial Extracorporeal Membrane Oxygenation: The EARLY-UNLOAD Randomized Clinical Trial. Circulation.

[17] Banning A.S., Sabaté M., Orban M., Gracey J., López-Sobrino T., Massberg S., Kastrati A., Bogaerts K., Adriaenssens T., Berry C. (2023). Venoarterial extracorporeal membrane oxygenation or standard care in patients with cardiogenic shock complicating acute myocardial infarction: The multicentre, randomised EURO SHOCK trial. EuroIntervention.

[18] Thiele H., Desch S., Freund A., Zeymer U. (2024). Why VA-ECMO should not be used routinely in AMI-Cardiogenic Shock. J. Heart Lung Transplant..

[19] Zeymer U., Freund A., Hochadel M., Ostadal P., Belohlavek J., Rokyta R., Massberg S., Brunner S., Lüsebrink E., Flather M. (2023). Venoarterial extracorporeal membrane oxygenation in patients with infarct-related cardiogenic shock: An individual patient data meta-analysis of randomised trials. Lancet.

[20] Møller J.E., Engstrøm T., Jensen L.O., Eiskjær H., Mangner N., Polzin A., Schulze P.C., Skurk C., Nordbeck P., Clemmensen P. (2024). Microaxial Flow Pump or Standard Care in Infarct-Related Cardiogenic Shock. N. Engl. J. Med..

[21] Keebler M.E., Haddad E.V., Choi C.W., McGrane S., Zalawadiya S., Schlendorf K.H., Brinkley D.M., Danter M.R., Wigger M., Menachem J.N. (2018). Venoarterial Extracorporeal Membrane Oxygenation in Cardiogenic Shock. JACC Heart Fail..

[22] Schmidt M., Burrell A., Roberts L., Bailey M., Sheldrake J., Rycus P.T., Hodgson C., Scheinkestel C., Cooper D.J., Thiagarajan R.R. (2015). Predicting survival after ECMO for refractory cardiogenic shock: The survival after veno-arterial-ECMO (SAVE)-score. Eur. Heart J..

[23] Amin F., Lombardi J., Alhussein M., Posada J.D., Suszko A., Koo M., Fan E., Ross H., Rao V., Alba A.C. (2021). Predicting Survival After VA-ECMO for Refractory Cardiogenic Shock: Validating the SAVE Score. CJC Open.

[24] Kanwar M.K., Shah P., Cascino T., Grinstein J., Goldstein D., Hernandez-Montfort J., Li S., Uriel N., Molina E., Cantor R. (2025). Impact of Preoperative Temporary Mechanical Circulatory Support on Durable LVAD Outcomes. JACC Heart Fail..

[25] Ahmed M.M., Jacobs J.P., Meece L.E., Jeng E.I., Bleiweis M.S., Cantor R.S., Singletary B., Kirklin J.K., Slaughter M.S. (2023). Timing and Outcomes of Concurrent and Sequential Biventricular Assist Device Implantation: A Society of Thoracic Surgeons Intermacs Analysis. Ann. Thorac. Surg..

[26] Chau V.Q., Coyle L., Pedersen R., Gallagher C., Graney N., Kukla L., Paliga R., Macaluso G.P., Pauwaa S., Cotts W.G. (2025). Analysis of outcomes in patients with HeartMate 3 with and without right ventricular assist device support. ESC Heart Fail..

[27] Willers A., Swol J., Buscher H., McQuilten Z., van Kuijk S.M.J., Cate H.T., Rycus P.T., McKellar S., Lorusso R., Tonna J.E. (2022). Longitudinal Trends in Bleeding Complications on Extracorporeal Life Support Over the Past Two Decades—Extracorporeal Life Support Organization Registry Analysis. Crit. Care Med..

[28] Singh S.K., Ning Y., Kurlansky P., Kaku Y., Naka Y., Takayama H., Sayer G., Uriel N., Masoumi A., Fried J.A. (2021). Impact of Venoarterial Extracorporeal Membrane Oxygenation Flow on Outcomes in Cardiogenic Shock. Asaio J..

[29] Cheng R., Hachamovitch R., Kittleson M., Patel J., Arabia F., Moriguchi J., Esmailian F., Azarbal B. (2014). Complications of Extracorporeal Membrane Oxygenation for Treatment of Cardiogenic Shock and Cardiac Arrest: A Meta-Analysis of 1,866 Adult Patients. Ann. Thorac. Surg..

[30] Patel H., Dupuis L., Bacchetta M., Hernandez A., Kanwar M.K., Lindenfeld J., Shah Z., Siddiqi H.K., Sinha S.S., Shah A.S. (2024). Three-year outcomes after bridge to transplantation ECMO—Pre- and post-2018 UNOS revised heart allocation system. J. Heart Lung Transplant..

[31] Guglin M., Zucker M.J., Bazan V.M., Bozkurt B., El Banayosy A., Estep J.D., Gurley J., Nelson K., Malyala R., Panjrath G.S. (2019). Venoarterial ECMO for Adults. J. Am. Coll. Cardiol..

[32] Pavlushkov E., Berman M., Valchanov K. (2017). Cannulation techniques for extracorporeal life support. Ann. Transl. Med..

[33] Geller B.J., Sinha S.S., Kapur N.K., Bakitas M., Balsam L.B., Chikwe J., Klein D.G., Kochar A., Masri S.C., Sims D.B. (2022). Escalating and De-escalating Temporary Mechanical Circulatory Support in Cardiogenic Shock: A Scientific Statement from the American Heart Association. Circulation.

[34] Moll V., Teo E.Y., Grenda D.S., Powell C.D., Connor M.J., Gartland B.T., Zellinger M.J., Bray H.B., Paciullo C.A., Kalin C.M. (2016). Rapid Development and Implementation of an ECMO Program. Asaio J..

[35] Corno A.F., Faulkner G.M., Harvey C. (2020). Mobile Extracorporeal Membrane Oxygenation. Asaio J..

[36] Bartos J.A., Frascone R., Conterato M., Wesley K., Lick C., Sipprell K., Vuljaj N., Burnett A., Peterson B.K., Simpson N. (2020). The Minnesota mobile extracorporeal cardiopulmonary resuscitation consortium for treatment of out-of-hospital refractory ventricular fibrillation: Program description, performance, and outcomes. eClinicalMedicine.

[37] Gottula A.L., Qi M., Lane B.H., Shaw C.R., Gorder K., Powell E., Danielson K., Ciullo A., Johnson N.J., Tonna J.E. (2024). Prehospital Ground and Helicopter-Based Extracorporeal Cardiopulmonary Resuscitation (ECPR) Reduce Barriers to ECPR: A GIS Model. Prehospital Emerg. Care.

[38] Julliard W., Teman N. (2020). Extracorporeal Membrane Oxygenation: How I Teach It. Ann. Thorac. Surg..

[39] Banfi C., Pozzi M., Brunner M.-E., Rigamonti F., Murith N., Mugnai D., Obadia J.-F., Bendjelid K., Giraud R. (2016). Veno-arterial extracorporeal membrane oxygenation: An overview of different cannulation techniques. J. Thorac. Dis..

[40] Mohamedali B., Tatooles A., Bhat G. (2014). Use of a single circuit to provide temporary mechanical respiratory and circulatory support in patients with LV apical thrombus and cardiogenic shock. Perfusion.

[41] Babu A. (2014). Techniques for Venoarterial Extracorporeal Membrane Oxygenation Support and Conversion to Temporary Left Ventricular Assist Device. Oper. Tech. Thorac. Cardiovasc. Surg..

[42] Keller S.P. (2019). Management of Peripheral Venoarterial Extracorporeal Membrane Oxygenation in Cardiogenic Shock. Crit. Care Med..

[43] Mahboub-Ahari A., Heidari F., Sadeghi-Ghyassi F., Asadi M. (2018). A systematic review of effectiveness and economic evaluation of Cardiohelp and portable devices for extracorporeal membrane oxygenation (ECMO). J. Artif. Organs.

[44] Merkle J., Djorjevic I., Sabashnikov A., Kuhn E.W., Deppe A.-C., Eghbalzadeh K., Fattulayev J., Hohmann C., Zeriouh M., Kuhn-Régnier F. (2017). Mobile ECMO—A divine technology or bridge to nowhere?. Expert Rev. Med. Devices.

[45] den Uil C.A., Akin S., Jewbali L.S., dos Reis Miranda D., Brugts J.J., Constantinescu A.A., Kappetein A.P., Caliskan K. (2017). Short-term mechanical circulatory support as a bridge to durable left ventricular assist device implantation in refractory cardiogenic shock: A systematic review and meta-analysis. Eur. J. Cardio-Thorac. Surg..

[46] Ezad S.M., Ryan M., Donker D.W., Pappalardo F., Barrett N., Camporota L., Price S., Kapur N.K., Perera D. (2023). Unloading the Left Ventricle in Venoarterial ECMO: In Whom, When, and How?. Circulation.

[47] Baldetti L., Gallone G. (2024). Left ventricular unloading and venting in veno-arterial extracorporeal membrane oxygenation: The importance of cardiogenic shock aetiology in guiding treatment strategies. ESC Heart Fail..

[48] Donker D.W., Brodie D., Henriques J.P., Broomé M. (2018). Left ventricular unloading during veno-arterial ECMO: A review of percutaneous and surgical unloading interventions. Perfusion.

[49] Singh-Kucukarslan G., Raad M., Al-Darzi W., Cowger J., Brice L., Basir M.B., O’nEill W.W., Alaswaad K., Eng M.H. (2021). Hemodynamic Effects of Left-Atrial Venous Arterial Extra-Corporeal Membrane Oxygenation (LAVA-ECMO). Asaio J..

[50] Jumean M., Pham D.T., Kapur N.K. (2015). Percutaneous bi-atrial extracorporeal membrane oxygenation for acute circulatory support in advanced heart failure. Catheter. Cardiovasc. Interv..

[51] Lim H.S. (2017). The Effect of Impella CP on Cardiopulmonary Physiology During Venoarterial Extracorporeal Membrane Oxygenation Support. Artif. Organs.

[52] Eliet J., Gaudard P., Zeroual N., Rouvière P., Albat B., Mourad M., Colson P.H. (2018). Effect of Impella During Veno-Arterial Extracorporeal Membrane Oxygenation on Pulmonary Artery Flow as Assessed by End-Tidal Carbon Dioxide. Asaio J..

[53] Schrage B., Becher P.M., Bernhardt A., Bezerra H., Blankenberg S., Brunner S., Colson P., Deseda G.C., Dabboura S., Eckner D. (2020). Left Ventricular Unloading Is Associated with Lower Mortality in Patients with Cardiogenic Shock Treated with Venoarterial Extracorporeal Membrane Oxygenation. Circulation.

[54] Grandin E.W., Nunez J.I., Willar B., Kennedy K., Rycus P., Tonna J.E., Kapur N.K., Shaefi S., Garan A.R. (2022). Mechanical Left Ventricular Unloading in Patients Undergoing Venoarterial Extracorporeal Membrane Oxygenation. J. Am. Coll. Cardiol..

[55] Saura O., Hékimian G., Del Marmol G., Lucenteforte M., de Chambrun M.P., Chommeloux J., Assouline B., Petit M., Juvin C., Gautier M. (2025). Effect of ECMO Flow Variations on Pulmonary Capillary Wedge Pressure in Patients with Cardiogenic Shock. J. Am. Coll. Cardiol..

[56] Yeo I., Axman R., Lu D.Y., Feldman D.N., Cheung J.W., Minutello R.M., Karas M.G., Iannacone E.M., Srivastava A., Girardi N.I. (2024). Impella Versus Intra-Aortic Balloon Pump in Patients with Cardiogenic Shock Treated with Venoarterial Extracorporeal Membrane Oxygenation: An Observational Study. J. Am. Heart Assoc..

[57] Lüsebrink E., Binzenhöfer L., Hering D., Sierra L.V., Schrage B., Scherer C., Speidl W.S., Uribarri A., Sabate M., Noc M. (2024). Scrutinizing the Role of Venoarterial Extracorporeal Membrane Oxygenation: Has Clinical Practice Outpaced the Evidence?. Circulation.

[58] Bharadwaj M.S., Bora V. (2024). Venoarterial ECMO Hemodynamics. StatPearls.

[59] Mikuz G., Höpfel-Kreiner I. (1982). Papillary mesothelioma of the tunica vaginalis propria testis. Virchows Arch..

[60] Møller J.E., Sionis A., Aissaoui N., Ariza A., Bělohlávek J., De Backer D., Färber G., Gollmann-Tepeköylu C., Mebazaa A., Price S. (2023). Step by step daily management of short-term mechanical circulatory support for cardiogenic shock in adults in the intensive cardiac care unit: A clinical consensus statement of the Association for Acute CardioVascular Care of the European Society of Cardiology SC, the European Society of Intensive Care Medicine, the European branch of the Extracorporeal Life Support Organization, and the European Association for Cardio-Thoracic Surgery. Eur. Heart J. Acute Cardiovasc. Care.

[61] Hussey P.T., von Mering G., Nanda N.C., Ahmed M.I., Addis D.R. (2022). Echocardiography for extracorporeal membrane oxygenation. Echocardiography.

[62] Douflé G., Roscoe A., Billia F., Fan E. (2015). Echocardiography for adult patients supported with extracorporeal membrane oxygenation. Crit. Care.

[63] Le Gall A., Follin A., Cholley B., Mantz J., Aissaoui N., Pirracchio R. (2018). Veno-arterial-ECMO in the intensive care unit: From technical aspects to clinical practice. Anaesth. Crit. Care Pain Med..

[64] Levy J.H., Alexander P.M.A., Wolberg A.S., McCarty O.J.T., Pusateri A.E., Bartz R.R., Bergmeier W., Cohen M.J., Connors J.M., Morrissey J.H. (2025). ECMO-induced coagulopathy: Strategic initiatives for research and clinical practice (a workshop report of the NHLBI). Blood Vessel. Thromb. Hemost..

[65] Rajsic S., Irsara C., Griesmacher A., Brunelli L., Breitkopf R., Innerhofer N., Eckhardt C., Treml B. (2025). Anticoagulation Monitoring During Extracorporeal Membrane Oxygenation: A Narrative Review. J. Cardiothorac. Vasc. Anesth..

[66] Rother R.P., Bell L., Hillmen P., Gladwin M.T. (2005). The Clinical Sequelae of Intravascular Hemolysis and Extracellular Plasma Hemoglobin. JAMA.

[67] Meegan J.E., Bastarache J.A., Ware L.B. (2021). Toxic effects of cell-free hemoglobin on the microvascular endothelium: Implications for pulmonary and nonpulmonary organ dysfunction. Am. J. Physiol. Cell. Mol. Physiol..

[68] Appelt H., Philipp A., Mueller T., Foltan M., Lubnow M., Lunz D., Zeman F., Lehle K. (2020). Factors associated with hemolysis during extracorporeal membrane oxygenation (ECMO)—Comparison of VA- versus VV ECMO. PLoS ONE.

[69] Aubron C., McQuilten Z., Bailey M., Board J., Buhr H., Cartwright B., Dennis M., Hodgson C., Forrest P., McIlroy D. (2019). Low-Dose Versus Therapeutic Anticoagulation in Patients on Extracorporeal Membrane Oxygenation: A Pilot Randomized Trial. Crit. Care Med..

[70] Vajter J., Volod O. (2025). Anticoagulation Management During ECMO: Narrative Review. JHLT Open.

[71] Schmidt M., Pellegrino V., Combes A., Scheinkestel C., Cooper D.J., Hodgson C. (2014). Mechanical ventilation during extracorporeal membrane oxygenation. Crit. Care.

[72] Rehder K.J., Alibrahim O.S. (2023). Mechanical Ventilation during ECMO: Best Practices. Respir. Care.

[73] Wang J.M., Gangavelli A.M., Tonna J.E., Magruder J.T., Zaaqoq A.M., Yalamuri S., Miller P.E., Jentzer J.C. (2025). Hyperoxia and End-Organ Complications Among Cardiogenic Shock Patients Supported by Venoarterial Extracorporeal Membrane Oxygenation. Crit. Care Med..

[74] Guarracino F., Baldassarri R., Brizzi G., Isirdi A., Landoni G., Marmiere M., Belletti A. (2025). Awake Venovenous Extracorporeal Membrane Oxygenation in the Intensive Care Unit: Challenges and Emerging Concepts. J. Cardiothorac. Vasc. Anesth..

[75] Tatooles A.J., Mustafa A.K., Joshi D.J., Pappas P.S. (2021). Extracorporeal membrane oxygenation with right ventricular support in COVID-19 patients with severe acute respiratory distress syndrome. JTCVS Open.

[76] Gurnani P.K., Michalak L.A., Tabachnick D., Kotwas M., Tatooles A.J. (2022). Outcomes of Extubated COVID and Non-COVID Patients Receiving Awake Venovenous Extracorporeal Membrane Oxygenation. Asaio J..

[77] Sniderman J., Monagle P., Annich G.M., MacLaren G. (2020). Hematologic concerns in extracorporeal membrane oxygenation. Res. Pract. Thromb. Haemost..

[78] Goel R., Roubinian N.H., Bembea M.M. (2023). Disentangling the causes of RBC loss and anemia in ECMO patients: Some questions answered?. Transfusion.

[79] Martucci G., Schmidt M., Agerstrand C., Tabatabai A., Tuzzolino F., Giani M., Ramanan R., Grasselli G., Schellongowski P., Riera J. (2022). Transfusion practice in patients receiving VV ECMO (PROTECMO): A prospective, multicentre, observational study. Lancet Respir. Med..

[80] E Tonna J. (2022). Patients receiving ECMO are special, but still only need a haemoglobin concentration of 7g/dL. Lancet Respir. Med..

[81] Pratt E.H., Pulsipher A.M., Moulton N.G., MacDonald A., Poehlein E., Green C.L., Rackley C.R. (2024). Association of RBC Transfusion Thresholds and Outcomes in Medical Patients with Acute Respiratory Failure Supported with Extracorporeal Membrane Oxygenation. Chest.

[82] Falk L., Sallisalmi M., Lindholm J.A., Lindfors M., Frenckner B., Broomé M., Broman L.M. (2019). Differential hypoxemia during venoarterial extracorporeal membrane oxygenation. Perfusion.

[83] Abrams D., Combes A., Brodie D. (2014). Extracorporeal Membrane Oxygenation in Cardiopulmonary Disease in Adults. J. Am. Coll. Cardiol..

[84] Tsangaris A., Alexy T., Kalra R., Kosmopoulos M., Elliott A., Bartos J.A., Yannopoulos D. (2021). Overview of Veno-Arterial Extracorporeal Membrane Oxygenation (VA-ECMO) Support for the Management of Cardiogenic Shock. Front. Cardiovasc. Med..

[85] Pinna S.M., Casasnovas I.S., Olmedo M., Machado M., Fernández M.J., Devesa-Cordero C., Galar A., Alvarez-Uria A., Fernández-Avilés F., Carreño J.G. (2023). Nosocomial Infections in Adult Patients Supported by Extracorporeal Membrane Oxygenation in a Cardiac Intensive Care Unit. Microorganisms.

[86] Ho M.-H., Lee J.J., Lai P.C.K., Li P.W.C. (2023). Prevalence of delirium among critically ill patients who received extracorporeal membrane oxygenation therapy: A systematic review and proportional meta-analysis. Intensiv. Crit. Care Nurs..

[87] Lewis K.M., Balas M.C.R., Stollings J.L.P., McNett M.R., Girard T.D.M., Chanques G., Kho M.E., Pandharipande P.P.M., Weinhouse G.L., Brummel N.E.M. (2025). A Focused Update to the Clinical Practice Guidelines for the Prevention and Management of Pain, Anxiety, Agitation/Sedation, Delirium, Immobility, and Sleep Disruption in Adult Patients in the ICU. Crit. Care Med..

[88] Cheng V., Abdul-Aziz M.-H., Roberts J.A., Shekar K. (2018). Optimising drug dosing in patients receiving extracorporeal membrane oxygenation. J. Thorac. Dis..

[89] Sherwin J., Heath T., Watt K. (2016). Pharmacokinetics and Dosing of Anti-infective Drugs in Patients on Extracorporeal Membrane Oxygenation: A Review of the Current Literature. Clin. Ther..

[90] Higa K.C., Mayer K., Quinn C., Jubina L., Suarez-Pierre A., Colborn K.P., Jolley S.E.M., Enfield K., Zwischenberger J., Sevin C.M. (2023). Sounding the Alarm: What Clinicians Need to Know about Physical, Emotional, and Cognitive Recovery After Venoarterial Extracorporeal Membrane Oxygenation. Crit. Care Med..

[91] Fernando S.M., Scott M., Talarico R., Fan E., McIsaac D.I., Sood M.M., Myran D.T., Herridge M.S., Needham D.M., Hodgson C.L. (2022). Association of Extracorporeal Membrane Oxygenation with New Mental Health Diagnoses in Adult Survivors of Critical Illness. JAMA.

[92] Dubb R., Nydahl P., Hermes C., Schwabbauer N., Toonstra A., Parker A.M., Kaltwasser A., Needham D.M. (2016). Barriers and Strategies for Early Mobilization of Patients in Intensive Care Units. Ann. Am. Thorac. Soc..

[93] Salna M., Abrams D., Brodie D. (2020). Physical rehabilitation in the awake patient receiving extracorporeal circulatory or gas exchange support. Ann. Transl. Med..

[94] Ohbe H., Jo T., Yamana H., Matsui H., Fushimi K., Yasunaga H. (2018). Early enteral nutrition for cardiogenic or obstructive shock requiring venoarterial extracorporeal membrane oxygenation: A nationwide inpatient database study. Intensiv. Care Med..

[95] Pardo E., Lescot T., Preiser J.-C., Massanet P., Pons A., Jaber S., Fraipont V., Levesque E., Ichai C., Petit L. (2023). Association between early nutrition support and 28-day mortality in critically ill patients: The FRANS prospective nutrition cohort study. Crit. Care.

[96] Park J., Heo E., Song I.-A., Cho J., Namgung H., Lee E., Lee E., Kim D.J. (2020). Nutritional support and clinical outcomes in critically ill patients supported with veno-arterial extracorporeal membrane oxygenation. Clin. Nutr..

[97] Dresen E., Naidoo O., Hill A., Elke G., Lindner M., Jonckheer J., De Waele E., Meybohm P., Modir R., Patel J.J. (2022). Medical nutrition therapy in patients receiving ECMO: Evidence-based guidance for clinical practice. J. Parenter. Enter. Nutr..

[98] Nair R.M., Chawla S., Mentias A., Saleem T., Vural A., Ko T., Rampersad P., Cremer P., Menon V. (2023). Glycemic patterns and impact of early hyperglycaemia in patients with cardiogenic shock on mechanical circulatory support. Eur. Heart J. Acute Cardiovasc. Care.

[99] Brahmbhatt D.H., Daly A.L., Luk A.C., Fan E., Billia F. (2021). Liberation from Venoarterial Extracorporeal Membrane Oxygenation: A Review. Circ. Heart Fail..

[100] Fried J.A., Masoumi A., Takeda K., Brodie D. (2020). How I approach weaning from venoarterial ECMO. Crit. Care.

[101] Aissaoui N., Luyt C.-E., Leprince P., Trouillet J.-L., Léger P., Pavie A., Diebold B., Chastre J., Combes A. (2011). Predictors of successful extracorporeal membrane oxygenation (ECMO) weaning after assistance for refractory cardiogenic shock. Intensiv. Care Med..

[102] Organ Procurement and Transplantation Network (OPTN) Adult Heart Approved Policy Language, Policy 6: Allocation of Hearts and Heart-Lungs. OPTN; Effective Date 2025-08-01. Published by U.S. Department of Health & Human Services. https://optn.transplant.hrsa.gov/media/2412/adult_heart_approved_policy_language.pdf..

[103] Vincent D.E., Moazami N., D’aLessandro D., Fraser J.F., Heinsar S., Roche E.T., Ayers B.C., Singh M., Langer N., Deshpande S.R. (2024). Pulsatile ECMO. JACC Basic Transl. Sci..

[104] Tipping C.J., Harrold M., Holland A., Romero L., Nisbet T., Hodgson C.L. (2016). The effects of active mobilisation and rehabilitation in ICU on mortality and function: A systematic review. Intensiv. Care Med..

[105] Abrams D., Madahar P., Eckhardt C.M., Short B., Yip N.H., Parekh M., Serra A., Dubois R.L., Saleem D., Agerstrand C. (2022). Early Mobilization during Extracorporeal Membrane Oxygenation for Cardiopulmonary Failure in Adults: Factors Associated with Intensity of Treatment. Ann. Am. Thorac. Soc..

[106] Schmack B., Hanke J.S., Schmitto J.D., Kühn C., Ruhparwar A. (2024). ECMO-TO-GO: Application of a portable on the body veno-arterial ECMO device in a bridge-to-transplantation setting. JHLT Open.

[107] Aultman J., Firstenberg M.S. (2019). Finding a bridge to somewhere: An ethical framework for veno-arterial extra- corporeal membrane oxygenation decisions. Advances in Extracorporeal Membrane Oxygenation.

[108] Wang S., Tao S., Zhu Y., Gu Q., Ni P., Zhang W., Wu C., Zhao R., Hu W., Diao M. (2025). AI-powered model for predicting mortality risk in VA-ECMO patients: A multicenter cohort study. Sci. Rep..

[109] Goldberg J. It Takes a Village to Determine the Origins of an African Proverb. NPR Goats and Soda. https://www.npr.org/sections/goatsandsoda/2016/07/30/487925796/it-takes-a-village-to-determine-the-origins-of-an-african-proverb.

